# Use of technology in end-of-life care discussions with COVID-19 patients: a narrative of a single institutional experience

**DOI:** 10.1080/10872981.2020.1830681

**Published:** 2020-10-12

**Authors:** Rucira Ooi, Setthasorn Zhi Yang Ooi

**Affiliations:** a Department of Obstetrics and Gynaecology, Princess of Wales Hospital, Mid Glamorgan, UK; b Cardiff University School of Medicine, University Hospital of Wales Main Building, Cardiff, UK

## Abstract

The art of communication is an important skill to have when breaking bad news or discussing end-of-life care with patients and their next of kin. Especially for COVID-19 patients, the discussions surrounding end-of-life care can be challenging and may present as unexpected news to the patient and to family members. In this letter, we explore the use of technology in end-of-life care discussions with COVID-19 patients.

## Letter

Since the declaration of a national lockdown (16 March 2020) in the UK, hospitals have tightened their policies on visitations and the strict need for personal protective equipment (PPE) use [1]. As such, many victims of the SARS-CoV-2 virus experience an isolated and lonely environment once admitted into the hospital. This included patients who are cognitively impaired, have hearing impairments, or have a language barrier. The challenges faced by both patients and family members surrounding end-of-life care discussions in a single, district general hospital with a catchment area of 600,000 people are explored.

In the first few weeks of the pandemic, we found it disheartening to turn family members and friends away from visiting patients in the ward. Although unpleasant, this was to limit the further spread of the virus through its airborne transmission. The only contact link between patients and their family members was through their doctors and nurses. The clinicians’ role as a bridging communicator evolved. The main form of contact for doctors to family members and/or next of kin during this period was the use of ward-based telephones. Consequently, victims of the virus had to die a lonely death.

As the number of SARS-CoV-2 patients increased throughout the UK, clinicians and staff members explored innovative and creative methods to make end-of-life care discussions more endurable. The use of technology such as iPads or similar tablets was adopted onto Coronavirus-confirmed wards and the intensive care unit to help ease the discussions around end-of-life care. We found that family members and friends were extremely appreciative of the introduction of these technologies. Although the experience is virtual, family members were able to see the patient and get directly involved in facilitating care surrounding difficult discussions regarding resuscitation and escalation of treatment. We found that patients also responded positively by being able to communicate with their friends and family members. This, too, helped improve patients’ mood, well-being, and mental health.

From a healthcare provider’s perspective, we found that there are many advantages to the use of ward-based tablets. These tablets provide all patients with a safe and reliable platform to communicate with their family members. This prevents discrimination against patients who are less proficient in the use of modern technology or do not have the technology for their utility or are too weak to operate their mobile devices. Using a standardized ward-based tablet for consultations as opposed to the use of patients’ mobile devices also acts in the best interest of infection control. Furthermore, the tablets provided at our hospital use an app (Attend Anywhere™) that prevents screen recording by the receiver (i.e. family members) during the video call, hence ensuring patient confidentiality [2].

The use of communication flashcards such as CARDMEDIC™ was also introduced at our hospital [3]. This, too, has eased conversations surrounding patients’ care and management received in the hospital. Patients found the flashcards user-friendly as it uses images and basic language to explain the care they will receive. In addition, the app provides multiple language translations that have aided difficult conversations with patients who may have a language barrier. Overall, clinicians found these flashcards useful as a communication tool, especially in the intensive care unit where face masks are worn. Face masks can prevent patients from lip-reading and turn muffled words into inaudible sounds.

The reality is that the SARS-CoV-2 virus has robbed many lives on a global scale, many of whom would still be alive if it was not for the virus. We found that the discussion of end-of-life care with patients affected by the virus often shocks them and their family members. These conversations are often daunting, distressing, and emotional for both the patients and their family members [4]. We can only imagine how agonizing it must be for patients who have to go through these conversations alone. Thankfully, the use of technology at our hospital has proven to allow patients the opportunity to connect with their family members in making these difficult decisions about their care. In addition to the benefits technology brings to the patients’ experience, it also increases the family members’ sense of empowerment and coping. This positively influences how family members experience and remember the period before and after the death of their loved ones.

Surprisingly, there are limited published articles on the availability and uptake of these technologies in hospitals. As we are all adapting to the ‘new normal’, we recommend hospitals worldwide to invest in these technologies to help facilitate conversations, especially those surrounding end-of-life care among patients infected with the SARS-CoV-2 virus.
